# Outcomes of patients on second‐ and third‐line ART enrolled in ART adherence clubs in Maputo, Mozambique

**DOI:** 10.1111/tmi.13490

**Published:** 2020-10-14

**Authors:** I. Finci, A. Flores, A. G. Gutierrez Zamudio, A. Matsinhe, E. de Abreu, S. Issufo, I. Gaspar, I. Ciglenecki, L. Molfino

**Affiliations:** ^1^ Médecins sans Frontières Maputo Mozambique; ^2^ Ministry of Health Maputo Mozambique; ^3^ Médecins sans Frontières Geneva Switzerland

**Keywords:** HIV care continuum, retention, differentiated care, adherence clubs, VL suppression, second‐ and third‐line ART patients

## Abstract

**Objectives:**

Adherence clubs (AC) offer patient‐centred access to antiretroviral therapy (ART) while reducing the burden on health facilities. AC were implemented in a health centre in Mozambique specialising in patients with a history of HIV treatment failure. We explored the impact of AC on retention in care and VL suppression of these patients.

**Methods:**

We performed a retrospective analysis of patients enrolled in AC receiving second‐ or third‐line ART. The Kaplan–Meier estimates were used to analyse retention in care in health facility, retention in AC and viral load (VL) suppression (VL < 1000 copies/mL). Predictors of attrition and VL rebound (VL ≥ 1000 copies/mL) were assessed using multivariable proportional hazards regression.

**Results:**

The analysed cohort contained 699 patients, median age 40 years [IQR: 35–47], 428 (61%) female and 97% second‐line ART. Overall, 9 (1.3%) patients died, and 10 (1.4%) were lost to follow‐up. Retention in care at months 12 and 24 was 98.9% (95% CI: 98.2–99.7) and 96.4% (95% CI: 94.6–98.2), respectively. Concurrently, 85.8% (95% CI: 83.1–88.2) and 80.9% (95% CI: 77.8–84.1) of patients maintained VL suppression. No association between predictors and all‐cause attrition or VL rebound was detected. Among 90 patients attending AC and simultaneously having VL rebound, 64 (71.1%) achieved VL resuppression, 10 (11.1%) did not resuppress, and 14 (15.6%) had no subsequent VL result.

**Conclusion:**

Implementation of AC in Mozambique was successful and demonstrated that patients with a history of HIV treatment failure can be successfully retained in care and have high VL suppression rate when enrolled in AC. Expansion of the AC model in Mozambique could improve overall retention in care and VL suppression while reducing workload in health facilities.

## Abstract (fr)

**Objectifs:**

Les clubs d'adhésion (CA) offrent un accès centré sur le patient à la thérapie antirétrovirale (ART) tout en réduisant la charge des établissements de santé. Les CA ont été mis en œuvre dans un centre de santé au Mozambique spécialisé dans les patients ayant des antécédents d'échec du traitement du VIH. Nous avons exploré l'impact des CA sur la rétention dans les soins et la suppression de la CV chez ces patients.

**Méthodes:**

Nous avons effectué une analyse rétrospective des patients enrôlés dans les CA sous ART de deuxième ou troisième intention. Les estimations de Kaplan‐Meyer ont été utilisées pour analyser la rétention dans les soins dans les établissements de santé, la rétention dans les CA et la suppression de la charge virale (CV) (CV <1000 copies/mL). Les prédicteurs de l'attrition et du rebond de la CV (VL ≥ 1000 copies/mL) ont été évalués à l'aide d'une régression à risques proportionnels multivariés.

**Résultats:**

La cohorte analysée contenait 699 patients, d'âge médian 40 ans [IQR: 35‐47], 428 (61%) femmes, 97% de traitement de deuxième intention. Dans l'ensemble, 9 (1,3%) patients sont décédés, 10 (1,4%) ont été perdus de vue. La rétention dans les soins à 12 et 24 mois était de 98,9% (IC95%: 98,2–99,7) et de 96,4% (IC95%: 94,6–98,2), respectivement. De même, 85,8% (IC95%: 83,1–88,2) et 80,9% (IC95%: 77,8–84,1) des patients ont maintenu une suppression de la CV. Aucune association entre les prédicteurs et l'attrition toutes causes ou le rebond de la CV n'a été détectée. Parmi 90 patients enrôlés dans les CA et ayant simultanément un rebond de la CV, 64 (71,1%) ont atteint une re‐suppression de la CV, 10 (11,1%) n'ont pas atteint une re‐suppression et 14 (15,6%) n'avaient pas de résultats de CV ultérieurs.

**Conclusion:**

La mise en œuvre des CA au Mozambique a été un succès et a démontré que les patients ayant des antécédents d'échec du traitement anti‐VIH peuvent être retenus avec succès dans les soins et ont un taux élevé de suppression de la CV lorsqu'ils sont enrôlés dans les CA. L'expansion du modèle CA au Mozambique pourrait améliorer la rétention globale dans les soins et la suppression de la CV tout en réduisant la charge de travail dans les établissements de santé.

## Introduction

In recent years, the scale‐up of antiretroviral therapy (ART) has decreased HIV‐related mortality [1] and resulted in HIV becoming a chronic and manageable disease conditioned upon lifelong treatment with strict adherence [2]. However, depending on the setting, patients experience different barriers towards remaining in care and adherent to ART [3, 4] such as long waiting times, cost of transport, competing life activities or stigma, among others. A recent study showed that after 5 years on ART, retention in care in sub‐Saharan Africa was 66.6% [5]. HIV services face the double challenge to provide timely initiations into ART for new patients while ensuring long‐term retention in care for a large number of patients enrolled in care [6]. To alleviate the problem, WHO endorsed differentiated models of service delivery [7] that provide patient‐centred care while simultaneously decreasing the patient load on healthcare facilities. Differentiated models of care vary in location, frequency, provider and type of services delivered [7, 8, 9], but prior reports on experiences with various models in sub‐Saharan Africa are primarily focused on stable patients on first‐line ART [10, 11]. Facility‐based ART adherence clubs, a differentiated model of care, were piloted in 2007 in South Africa [12], and the results of the pilot showed 97% retention in care for adherence club (AC) patients vs. 85% for patients who remained in routine care [13]. The ART refill visits are separated from clinical consultations, so these result in decongestion of health facilities and reductions in waiting time. Since AC are managed by healthcare workers, it is possible to detect early complications and perform quick clinical follow‐ups.

This article describes outcomes of patients enrolled in facility‐based AC at a Médecins sans Frontières (MSF)‐supported HIV clinic in Maputo, which provides care to patients with history of treatment failure and/or advanced HIV disease.

## Methods

### Setting

The Centro de Referência de Alto‐Maé (CRAM) was established in 2009 as a collaboration between the Mozambican Ministry of Health and MSF in Chamanculo Health District, in Maputo, Mozambique. CRAM is an intermediate centre between primary healthcare and referral hospitals, providing access to specialised ambulatory care for HIV‐infected patients with comorbid conditions or disease‐related complications. CRAM offers second‐ and third‐line ART for adults and children, chemotherapy for HIV‐associated Kaposi sarcoma, diagnosis and treatment of opportunistic infections, management of side effects of ART and hepatitis C treatment. As of March 2018, CRAM was treating 2577 patients on ARTs, of whom 1587 (61.5%) were on second‐line and 65 (2.5%) on third‐line ARTs. The ART regimens were standardised as recommended by the Ministry of Health: protease inhibitor‐based second‐line and integrase inhibitor‐based third‐line regimens.

### ART adherence clubs

AC were first implemented in CRAM in October 2015. Originally, patients older than 18 years who were on the same ARV regimen for minimum 6 months with undetectable viral loads were invited to enter AC. In late 2016, additional entry criterion for AC of CD4 > 250 cells/µL was introduced due to the increased need of close clinical follow‐up in the advanced disease population. Groups of 20 participants were formed and convened in CRAM once per month for 45 min in the early morning for patient convenience. Over time, a total of 48 clubs were formed, which resulted in two or at times three club sessions in one morning. While a handful of disease‐specific clubs were formed, we limited our analysis to groups of patients receiving second‐ or third‐line regimens.

Monthly group sessions were led by a lay counsellor, and different health‐related topics were discussed. Patients also underwent brief health screenings, and those with any symptoms received same‐day medical consultations. Medicines were pre‐packaged and distributed by the pharmacist during the sessions. Patients were required to attend scheduled AC sessions; however, sending a peer or visiting the health centre within five days of the session was acceptable, unless done two or more consecutive times. Patients completed full semi‐annual clinical consultations, and VL was measured four months after AC entry, and if suppressed (VL < 1000 copies/mL), every 12 months thereafter. If VL was detectable but below 1000 cp/mL, patients received enhanced adherence counselling (EAC). Beginning in late 2016, patients who had a measured VL ≥ 1,000 cp/ml exited the AC, at least temporarily, and received intensive EAC, which included adherence support using motivational psychology together with medical management by clinical staff. After successful resuppression, these patients could return to AC, or if failing to suppress, they would exit AC permanently to investigate ART resistance. In addition to VL ≥ 1000 copies/mL, patients would exit the clubs if they developed a comorbidity requiring medical follow‐up, or if they did not attend club sessions regularly (e.g. sending peers or not attending at all in two consecutive visits). Patients who exited the clubs continued to be treated at CRAM per routine.

### Statistical analysis

Patient‐level data were collected and entered in FUCHSIA software (version 1.7.1; Epicentre, France). The follow‐up period began from the date of AC enrolment and ended at the date of analysis closure (31 March 2018) or the date of the outcome in the case of death, loss to follow‐up (LTFU) or transfer to another health centre. LTFU was defined as patients without a recorded visit to the clinic for more than 120 days. In the retention in care analysis, where the focus was on the time the patient spent in the health centre, the outcome of interest was LTFU in the health centre or death. In the analysis of retention in AC, where the focus was on the time the patient spent in the AC, the outcome of interest was time to exit from AC, LTFU in the AC or death. In both cases, censoring occurred in the case of transfer to a different health centre or analysis closure. Viral rebound was defined as the first viral load result of ≥1000 copies/mL after enrolling into AC.

Patient baseline characteristics were described using median and interquartile ranges for continuous variables and frequencies and proportions for categorical variables.

Analysis of retention in care, retention in AC and VL suppression were conducted using the Kaplan–Meier method. VL suppression was analysed by monitoring time to first VL rebound.

Univariable and multivariable Cox proportional hazards models were used to assess predictors of attrition and VL rebound, adjusting for baseline factors: age, sex, time on ART and ART regimen. All variables were used to build the model. Proportional hazards assumptions were tested using Schoenfeld’s global test. All statistical analyses were performed using R statistical software v3.4.2 [14].

### Ethics statement

This research was based on the analysis of routinely collected data and as such did not require review by Mozambican Ethics Review Board. Additionally, it fulfilled exemption criteria set by the Médecins Sans Frontières Ethics Review Board (ERB) for *a posteriori* analyses of routinely collected clinical data and thus did not require MSF ERB. Finally, informed consent was not sought from individual patients as this study was a retrospective cohort analysis of routinely collected data.

## Results

### Baseline characteristics

A total of 699 patients on second‐ and third‐line ART were enrolled into AC between October 2015 and March 2018, contributing 1123 person‐years. The median age of participants enrolling in AC was 40 (IQR, 35–47) years; 428 (61.2%) patients were female and median time on ART was 6.8 years (IQR, 4.4–9.9). Most patients (680, 97.3%) were on second‐line ART, and 19 (2.7%) patients were on third‐line ART (Table 1).

**Table 1:** Baseline characteristics of patients enrolled into adherence clubs

*N* = 699	*N* (%)
Gender, *n* (%)	
Males	271 (38.8%)
Females	428 (61.2%)
Age at club start	
Median, IQR	40 (35–47)
Categorical, *n* (%)	
≤24	36 (5.1%)
25–44	443 (63.4%)
45+	220 (31.5%)
ART regimen	
2^nd^ line	680 (97.3%)
3^rd^ line	19 (2.7%)
Median time on ART in years, IQR NA = 41 (5.9%)	6.8 (4.4–9.9)
Categories, time on ART	
<3 years	74 (10.6%)
3–6 years	192 (27.5%)
6–9 years	162 (23.2%)
>9 years	230 (32.9%)
NA = 41 (5.9%)	
Median time on second‐line ART in years, IQR	1.4 (0.9–2.3)
Median time on third‐line ART in years, IQR	0.9 (0.8–1.5)
Overall outcomes (care)	
In care	672 (96.1%)
LTFU	10 (1.4%)
Death	9 (1.3%)
Transfer out	8 (1.1%)

### Retention in care

**Figure 1:**
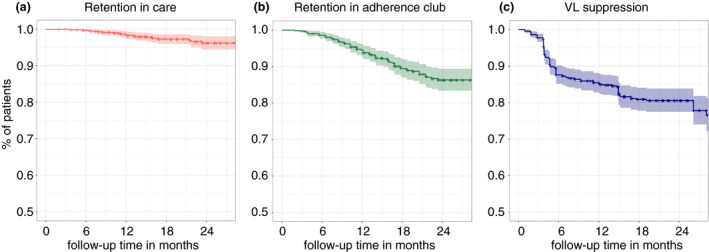
Kaplan–Meier survival analysis of retention and viral load suppression of club patients. Dark line shows survival curve; the light‐coloured area around it shows 95% confidence interval, censoring events are shown as +; A – retention in care for club patients – reflects the retention in the healthcare facility (CRAM); B – retention in adherence club; C – viral load suppression of club patients – measured as VL < 1000.

Retention in care was 98.9% (95% CI: 98.2–99.7), 97.4% (95% CI: 96.1–98.8) and 96.4 (95% CI: 94.6–98.2) at 12, 18 and 24 months, respectively (Table 2, Figure 1). During the analysis period, 9 (1.3%) patients died, 10 (1.4%) were LTFU, and 8 (1.1%) were transferred to another health facility. Baseline factors used in the multivariable analysis did not show any association with attrition (LTFU or death) from care (Table S1).

**Table 2:** Kaplan–Meier survival analysis for retention in care, retention in adherence clubs and VL suppression

Follow‐up time (months)	Retention in care *N* = 699			Retention in AC *N* = 699			Suppression of VL *N* = 674		
	*N* risk	*N* event	% [95% CI]	*N* risk	*N* event	% [95% CI]	*N* risk	*N* event	% [95% CI]
3	699	0	100 [100–100]	697	2	99.7 [99.3–100]	657	15	97.8 [96.7–98.9]
6	691	1	99.9 [99.6–100]	686	7	98.7 [97.9–99.6]	583	68	87.6 [85.1–90.1]
12	553	6	98.9 [98.2–99.7]	528	32	93.8 [91.9–95.6]	516	12	85.8 [83.1–88.2]
18	361	7	97.4 [96.1–98.8]	336	23	88.6 [85.9–91.3]	304	22	81.2 [78.1–84.4]
24	191	3	96.4 [94.6–98.2]	171	11	84.7 [81.4–88.2]	173	1	80.9 [77.8–84.1]

### Retention in Adherence Clubs

Retention in AC for the same patients at 12, 18 and 24 months was 93.8% (95% CI: 91.9–95.6), 88.6% (95% CI: 85.9–91.3) and 84.7% (95% CI: 81.4–88.2), respectively (Table 2, Figure 1). During the analysis period, 82 (12%) patients exited AC. Of these, 30 (36.6%) experienced VL rebound (>1000 copies/mL). Other recorded reasons for patients exiting were as follows: insufficient attendance (24; 29.2%), death (6; 7.3%), transfer to another health centre (6; 7.3%), LTFU (5; 6.1%), new comorbidity (5; 6.1%) and other reasons (6; 7.3%). Of 82 patients who exited AC, 55 (67.1%) were retained in care after they returned to normal care. For those patients, the main reasons for exiting AC were VL rebound (24; 43.6%), insufficient attendance (21; 38.1%), new comorbidity (5; 9.1%) and other reasons (6; 10.9%). Ninety patients (12.8%) did not exit AC despite having VL rebound.

### VL suppression

Among 699 patients in AC, 670 (96.1%) had at least one VL result after entering AC; together, they contributed 969 person‐years of follow‐up to the analysis. From these, 120 (15.5%) experienced VL rebound during the follow‐up period, with a VL rebound rate of 12.4 per 100 person‐years. Rates of VL suppression were 85.8% (95% CI: 83.1–88.2), 81.2% (95% CI: 78.1–84.4) and 80.9% (95% CI: 77.8–84.1) at 12, 18 and 24 months (Table 2, Figure 1). The adjusted multivariable analysis did not reveal significant associations between any analysed risk factor and VL rebound (Table S2). The VL testing uptake at median time of 4 months after entry into AC was 96.1% with 86.7% of the VL results < 1000 copies/mL. After 16 months in AC, the uptake was 84.8% with 93.2% having continued VL suppression (Table 3). At the first VL testing after club entry, 89 (13.3%) patients had a VL ≥ 1000 copies/mL, of these in the next VL test, 79 (88.8%) had a result, with 57 (72.5%) having resuppressed their VL. Further subanalysis showed that of the latter, 52 patients never exited AC and were still active in AC at the end of analysis. Therefore, for patients who never left the AC despite the first VL result of ≥1000 copies/mL, the second VL resulted in 80% VL resuppression, 10.8% VL rebound, and 9.2% did not have the second VL result. Overall, from 90 patients who did not exit the AC despite having VL rebound, 64 (71.1%) had VL resuppression by the end of the analysis period, 10 (11.1%) did not resuppress, and 14 (15.6%) did not have additional VL result. Of 64 patients who resuppressed VL while staying in the club, 30 (46.9%) attended a form of EAC sessions in order to enhance adherence, while 34 (53.1%) did not have an EAC session. For most cases, not having EAC session was due to delay in VL result reporting, and by the time VL was recorded and acted upon, these patients had VL resuppression.

**Table 3:** VL uptake and suppression after 4 months and 16 months in AC

AC follow‐up time (months)	4	16
Patients followed, *N*	697	381
Patients with VL, *N* (%)	670 (96.1%)	323 (84.8%)
VL < 1000	581 (86.7%)	301 (93.2%)
VL ≥ 1000	89 (13.3%)	22 (6.8%)

## Discussion

We describe implementation and analyse outcomes in ART adherence clubs as a model of care for patients on second‐ and third‐line ART in Mozambique. These analyses demonstrate that AC patients’ outcomes were remarkable; that is, overall retention in care was very high, 96.4% after 24 months. When considering that all the patients analysed had a history of prior treatment failure, the retention in care is even more encouraging.

The results of this study are in line with or slightly higher compared with previous findings of high retention in care of AC patients. In similar studies, retention in care for AC patients after 12 months was 97% in the pilot study in South Africa [13], 94.4% in Swaziland [15] and 94.8% for AC patients following VL resuppression [16]. Recently, a randomised control trial in South Africa showed higher retention in care of AC patients compared with the control group [17] reinforcing the notion that AC increase the retention in care. Retention in the AC model of care was also high, 93.8% after 12 and 84.7% after 24 months, in line with a similar study from South Africa where retention in AC at 12 months was 92% [16], and from Swaziland, 90.4% [15]. Such high retention in AC suggests active buy‐in of the AC model from a large majority of patients. For some of the patients who exited AC due to non‐attendance, the AC model of care was probably not adapted to their specific needs, and other differentiated models such as fast‐track ART distribution might have been more suitable. The fact that we observed higher retention in care compared with retention in AC would indicate that despite exiting the clubs, patients continued to attend the health facility and stayed in care. Therefore, the AC model was flexible, where patients could move between the AC, standard clinical care and other models of care based on their needs.

The main reason for exiting AC was VL rebound. At 18 months, VL suppression was 80.9%, similar to another report from South Africa describing patients with a history of unsuppressed VL of 75% [16]. The VL rebound rate in our study was 12.4 per 100 person‐years, which is also comparable to other reports on second‐line patients [18]. When compared to VL rebound at 4 and 16 months upon AC enrolment, the rate was slightly higher in our study compared with AC in South Africa, though the latter were comprised of patients on first‐line regimens [19]. An important enabling factor that enhances adherence and retention is the peer support that is created inside the AC, where patients bond with each other, share experiences and offer mutual support and encouragement [20, 21]. In addition, patients see club membership as a privilege and achievement [22], which also can facilitate adherence, as patients do not want to lose this privilege and exit from AC [21]. Additionally, our sessions were held early in the morning, which allowed patients to continue their daily routine activities without disruption. Simultaneously, for the health facility, having AC early in the morning resulted in overall clinic decompression and enabled focus on different groups of patients. Every morning, two or sometimes three AC sessions were held, and afterwards, healthcare workers continued working in the health centre per routine. Decompression due to AC and its impact on the health facility were not the focus of the study, but other studies have shown the cost‐effectiveness of AC in this manner [23].

A number of patients who had a VL rebound nevertheless stayed in the AC due to health facility staff not strictly applying the AC algorithm, and their experience is instructive. By the end of the analysis, 71% (64 out of 90) patients resuppressed their VL while continuing to participate in AC. This outcome is encouraging, and it is worth considering AC as a model of care for patients with VL rebound in the future. Moreover, as half of the patients resuppressed VL without attending EAC, it further suggests that peer support was one of the enabling factors for resuppression. There is a clear demand for differentiated models of care that would focus on unstable patients [9], and in AC model of care, unstable patients could benefit from peer support.

The specific focus on patients with a history of treatment failure and receiving second‐ and third‐line ART in this study arises from CRAM’s unique position in the health setting of Maputo. Even so, these results demonstrate that patients on second‐ and third‐line ART, with a history of treatment failure can be just as stable as patients on first‐line ART. In addition, the results do not imply that second‐ and third‐line patients should be separated from first‐line patients, and where healthcare workers have been trained to manage different ART lines, these different groups of patients could be mixed. In CRAM, due to medication stock challenges, the clubs were meeting once per month, whereas in other settings, they were reconvening less regularly, for example every 2, 3 or 6 months [15, 24, 25]. Having less frequent sessions, when medication stocks allow, like in South Africa where patients reconvened every 6 months, proved to be more convenient for the patients [25]. Having longer intervals between club meetings would simultaneously create new slots for early morning clubs and enable recruiting of new patients.

Mozambique has a low national rate of retention in care and adherence to ART; that is, the 12‐month retention rate in 2017 was 70% [26]. Hence, innovative strategies such as adherence clubs can help to improve national retention in care for new and long‐term HIV patients. Much can be learned from more than 10 years of experience with AC in South Africa [27]. The AC model is being rolled out by the Ministry of Health of Mozambique, and second‐line patients from CRAM were transferred to other healthcare centres where new AC have been formed based on our model. However, choosing the right model of care should be carefully considered depending on the setting. While this study focused on an urban setting, other reports have shown high retention in care in rural Mozambique (Tete province) after implementing community ART groups [28]. AC in our setting operated within the health facility, but community‐based AC that can further decompress health facilities has also demonstrated high retention in care in South Africa [29]. To truly provide patient‐centred care would mean offering different models of care for patients to choose for themselves, as was implemented in a region in Swaziland where three differentiated models of care were offered and the flexibility to move between different models did not affect retention [15].

This study has several limitations. Our data were not collected for an a priori analysis, and as a result, some baseline characteristics such as CD4 count at the AC start were missing and could not be considered for multivariable analysis. In addition, the number of outcomes of interest was low making attrition and VL rebound risk factor analysis limited. We also had no similar control by which to compare retention and VL suppression. Finally, calculating VL suppression depends on the data on VL testing, and since the testing was done mainly at 4, 16 and 28 months after enrolment to AC, not many patients had a third VL result, the measurement of VL suppression at 24 months may be less reliable.

## Conclusion

The AC model of care piloted in Mozambique improves adherence and long‐term retention in care among clinically stable patients on second‐ and third‐line ART, decreasing the burden of stable ART patients while optimising health resources that can be focused on other complicated patients. AC foster active buy‐in from patients and facility staff by addressing the key challenges that stable ART patients face in health facilities. It also shows that patients with a history of treatment failure can have good retention in care and adherence to ART treatment, and that HIV patients with significant comorbidities can be integrated into AC. Wider utilisation of our AC model in Mozambique and other similar contexts is justified.

## Supporting information


**Table S1.** Unadjusted and adjusted Cox regression risk factors for attrition from care (death or LTFU).
**Table S2.** Unadjusted and adjusted Cox regression risk factors for VL rebound (VL ≥ 1000 cp/mL).
