# Supplementary material for: Outcomes of patients on second‐ and third‐line ART enrolled in ART adherence clubs in Maputo, Mozambique
Source: Trop Med Int Health. 2020 Oct 14;25(12):1496–502. doi: 10.1111/tmi.13490 (PMC7756444; doi:10.1111/tmi.13490)
Supplement: Supplementary file 1 — Table S1. Unadjusted and adjusted Cox regression risk factors for attrition from care (death or LTFU). Table S2. Unadjusted and adjusted Cox regression risk factors for VL rebound (VL ≥ 1000 cp/mL). [file TMI-25-1496-s001.docx]

**Appendix**

**Table S1**. Unadjusted and adjusted Cox regression risk factors for attrition from care (death or LTFU)

|  | | **N event (%)** | **Unadjusted estimates** | | | **Adjusted model n=699** | | |  |
| --- | --- | --- | --- | --- | --- | --- | --- | --- | --- |
|  |  |  | **HR** | **95% CI** | **p** | **HR** | **95% CI** | **p** |  |
| **Gender** | Female | 13 (3.0) | ref |  |  | 1 |  |  | |
|  | Male | 6 (2.2) | 0.68 | 0.26-1.79 | 0.436 | 0.75 | 0.28-2.0 | 0.57 | |
| **Age-group years†** | ≤ 24 | 0 (0.0) |  |  |  |  |  |  | |
|  | 25-44 | 13 (2.9) |  |  |  |  |  |  | |
|  | ≥ 45 | 6 (2.7) |  |  |  |  |  |  | |
| **Time on ART**  **(NA=41)** | 0-3 | 1 (1.4) | ref |  |  | 1 |  |  | |
|  | 3-6 | 5 (2.6) | 1.70 | 0.20-14.62 | 0.63 | 1.7 | 0.20-14.6 | 0.63 | |
|  | 6-9 | 3 (1.9) | 1.07 | 0.11-10.38 | 0.95 | 1.05 | 0.11-10.2 | 0.97 | |
|  | ≥ 9 | 10 (4.3) | 2.49 | 0.32-19.56 | 0.39 | 2.67 | 0.33-21.1 | 0.36 | |
| **Regimen†** | 2^nd^ line | 19 (2.8) |  |  |  |  |  |  | |
|  | 3^rd^ line | 0 (0) |  |  |  |  |  |  | |

**†**these factors had categories with 0 events, so HR and CI cannot be calculated

**Table S2**. Unadjusted and adjusted Cox regression risk factors for VL rebound (VL≥1000 cp/ml)

|  | | **N event (%)** | **Unadjusted estimates** | | | **Adjusted model n=674** | | |
| --- | --- | --- | --- | --- | --- | --- | --- | --- |
|  |  |  | **HR** | **95% CI** | **p** | **HR** | **95% CI** | **p** |
| **Gender** | Female | 73 (17.5) | ref |  |  | ref |  |  |
|  | Male | 47 (18.2) | 1.02 | 0.71-1.48 | 0.90 | 1.08 | 0.74-1.59 | 0.69 |
| **Age-group years** | ≤ 24 | 4 (12.9) | ref |  |  | ref |  |  |
|  | 25-44 | 83 (19.4) | 1.71 | 0.63-4.67 | 0.30 | 1.73 | 0.63-4.76 | 0.29 |
|  | ≥ 45 | 33 (15.3) | 1.29 | 0.46-3.65 | 0.63 | 1.21 | 0.43-3.45 | 0.72 |
| **Time on ART**  **(NA=40)** | 0-3 | 7 (10.0) | ref |  |  | ref |  |  |
|  | 3-6 | 31 (16.5) | 1.73 | 0.78-3.99 | 0.19 | 1.80 | 0.79-4.09 | 0.16 |
|  | 6-9 | 30 (19.2) | 1.88 | 0.86-4.41 | 0.13 | 1.95 | 0.85-4.34 | 0.11 |
|  | ≥ 9 | 43 (19.5) | 1.96 | 0.92-4.53 | 0.10 | 2.16 | 0.96-4.85 | 0.06 |
| **Regimen** | 2^nd^ line | 117 (17.8) | ref |  |  | ref |  |  |
|  | 3^rd^ line | 3 (17.6) | 1.15 | 0.37-3.64 | 0.81 | 1.09 | 0.34-3.48 | 0.88 |
